# Maf1, a New Player in the Regulation of Human RNA Polymerase III Transcription

**DOI:** 10.1371/journal.pone.0000134

**Published:** 2006-12-27

**Authors:** Jaime H. Reina, Teldja N. Azzouz, Nouria Hernandez

**Affiliations:** Center for Integrative Genomics (CIG), University of Lausanne, Lausanne, Switzerland; University of Hong Kong, China

## Abstract

**Background:**

Human RNA polymerase III (pol III) transcription is regulated by several factors, including the tumor suppressors P53 and Rb, and the proto-oncogene c-Myc. In yeast, which lacks these proteins, a central regulator of pol III transcription, called Maf1, has been described. Maf1 is required for repression of pol III transcription in response to several signal transduction pathways and is broadly conserved in eukaryotes.

**Methodology/Principal Findings:**

We show that human endogenous Maf1 can be co-immunoprecipitated with pol III and associates *in vitro* with two pol III subunits, the largest subunit RPC1 and the α-like subunit RPAC2. Maf1 represses pol III transcription *in vitro* and *in vivo* and is required for maximal pol III repression after exposure to MMS or rapamycin, treatments that both lead to Maf1 dephosphorylation.

**Conclusions/Significance:**

These data suggest that Maf1 is a major regulator of pol III transcription in human cells.

## Introduction

RNA polymerase III (pol III) is responsible for the transcription of various short genes encoding untranslated RNAs involved in the maturation of other RNA molecules and in protein biosynthesis. These untranslated RNAs are essential for cell growth and proliferation, and are often abundant and stable. Consequently, pol III transcription is highly regulated, being high in rapidly dividing cells, which need to duplicate a large number of pol III transcripts in a limited time, and low in resting cells, where the demand for pol III activity is probably largely limited to the replacement of slowly decaying pol III RNAs (see [1], [2], and references therein). Moreover, pol III transcription is rapidly inhibited after a number of stresses that arrest cell growth and/or division, such as DNA damage or rapamycin treatment. In human cells so far, the main known pol III regulation mechanisms involve tumor suppressors and proto-oncogenes whose first identified transcription functions were in the regulation of pol II promoters [2], [3].

Pol III promoters use dedicated transcription factors as well as factors also used by pol II promoters. In human cells and their viruses, there are three main types of pol III promoters, the gene-internal type 1 promoter of the 5S small ribosomal RNA gene, the gene-internal type 2 promoters of the transfer RNA (tRNA) or Adenovirus 2 (Ad2) VAI genes, and the gene-external type 3 promoters of, for example, the U6 snRNA, 7SK, and H1 genes (see [1], [4], [5] for reviews). On type 1 promoters, the initial binding of the zinc protein TFIIIA allows the successive recruitment of the multisubunit complex TFIIIC and the Brf1-TFIIIB activity, composed of the TATA box binding protein TBP, the TFIIB-related factor Brf1, and the SANT domain protein Bdp1. Type 2 promoters recruit the same factors except that in this case, the promoter elements recruit TFIIIC directly, without the help of TFIIIA. The core type 3 promoters are composed of a proximal element (PSE) and a TATA box that recruit, respectively, the multisubunit complex SNAP_c_ and the TBP component of Brf2-TFIIIB, an activity similar to Brf1-TFIIIB except that Brf1 is replaced by another TFIIB-related factor referred to as Brf2 (see [1], [4], [5] for reviews).

Pol III transcription in mammalian cells is repressed by the tumor suppressors Rb and P53, which both affect transcription from all three types of pol III promoters (see [2], [3], [6] for reviews). Rb down-.regulates type 1 and 2 promoters by binding through its large pocket domain to Brf1-TFIIIB and preventing interactions with TFIIIC and pol III that are presumably required for efficient transcription complex assembly [7]–[9]. At type 3 promoters, it interacts with SNAP_c_ on DNA and inactivates transcription at a step subsequent to pol III recruitment [10], [11]. The mechanisms by which P53 down-regulates transcription are less well characterized but the protein is known to associate with TBP and SNAP_c_ [12]–[14].

Recently, a key player in the down-regulation of pol III transcription after stress or at quiescence was discovered in *Saccharomyces cerevisiae* [15]. This repressor, referred to as Maf1, was originally identified in *S. cerevisiae* by the isolation of a temperature-sensitive mutation, *maf1-1*, that affected tRNA suppressor efficiency and interacted genetically with pol III [16]. In *maf1-1* cells, tRNA levels were elevated, and pol III transcription was much more active in extracts from such cells than in extracts from wild-type cells, suggesting that Maf1 represses pol III transcription [17]. A key advance was the subsequent characterization of Maf1 as a common component of at least three signaling pathways that lead to pol III transcription repression, the secretory defect signaling pathway, the target of rapamycin (TOR) signaling pathway, and the DNA damage signaling pathway ([15], see [18] for a review).

Recent work [19], [20] has considerably advanced our understanding of the role of Maf1 (see [21] for a review). In actively growing yeast cells, Maf1 is present in both the nucleus and the cytoplasm, and a large fraction of Maf1 is phosphorylated, at least in part by PKA, whose activity counteracts Maf1 repression [22]. Upon exposure of the cells to various stresses, Maf1 is dephosphorylated in a manner dependent on PP2A and translocates to the nucleus, where it occupies pol III promoters as determined by genome-wide chromatin immunoprecipitations [19], [20]. The dephosphorylated form of Maf1 can associate with pol III, and recombinant Maf1 can bind to a protein fragment corresponding to the N-terminal 235 amino acids of the largest pol III subunit [19]. Yet, in chromatin immunoprecipitations, the increase in Maf1 signal at pol III promoters after stress is accompanied by a decrease in pol III signal, perhaps reflecting a change in polymerase conformation upon Maf1 binding that prevents efficient crosslinking to DNA [20]. *In vitro*, Maf1 is capable of preventing assembly of a transcription complex by binding to Brf1, suggesting that in addition to inhibiting transcription through binding to pol III, Maf1 can also prevent the assembly of new transcription complexes [23]. These results suggest that Maf1 is a central player in transduction pathways that lead to repression of pol III transcription, and give the first indications of the mechanisms by which Maf1 inhibits pol III transcription.

The discovery of yeast Maf1, and the observation that Maf1 is conserved in other species [17], raise the important question of whether Maf1 plays a similarly central role in pol III repression in mammalian cells. Here we show that human Maf1 is a repressor of pol III transcription both *in vitro* and *in vivo*. It down-regulates transcription non only from type 1 and 2 promoters, but also from type 3 promoters, which do not exist in yeast, and can do so in non-transformed cells as well as in transformed cells that do not respond to some stress signals. Maf1 associates with pol III *in vivo* and *in vitro*. Of all the individual subunits of Brf1-TFIIIB, Brf2-TFIIIB, SNAP_c_, and pol III, Maf1 associates weakly with Brf1 and RPC1, the largest subunit of pol III, and strongly with RPAC2. Like the yeast protein, human Maf1 is phosphorylated and becomes largely dephosphorylated after stress, and it is the dephosphorylated form of Maf1 that associates with pol III. The results suggest that Maf1 is a major player in repression of pol III transcription in mammalian cells.

## Results

### Maf1 represses transcription from type I, II, and III pol III promoters *in vitro*

**Figure 1:**
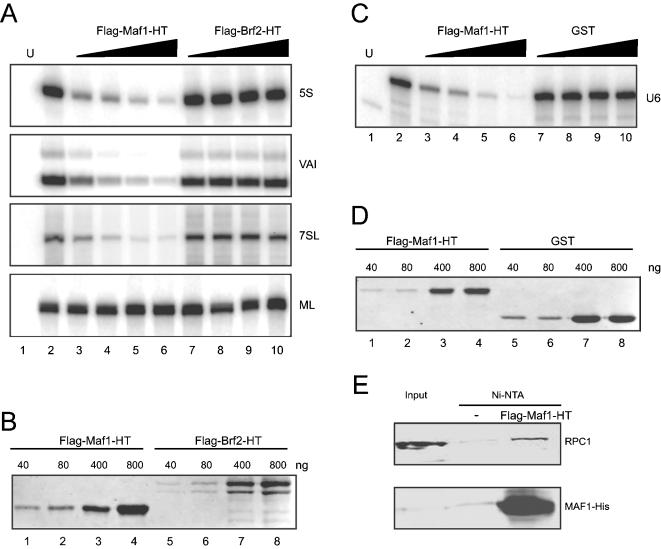
Maf1 represses transcription from type I, II, and III pol III promoters *in vitro.* A) Maf1 represses transcription from the 5S, VAI, and 7SL pol III promoters, but not the Ad2 ML pol II promoter, *in vitro*. 40, 80, 400, and 800 ng of bacterially produced Maf1 (lanes 3–6) or similar amounts of Brf2 (lanes 7–10) were added to transcription reactions identical to that shown in lane 2. Lane 1 shows unprogrammed transcription extract. B) SDS-polyacrylamide gel stained with coomassie blue indicating the amounts of recombinant Maf1 and Brf2 used in the *in vitro* transcription experiment described in A. C) Maf1 represses transcription from the U6 promoter *in vitro*. The lanes are as in A, except that the HeLa cell extract was programmed with the U6 promoter and that increasing amounts of GST rather than Brf2 were added as control. D) SDS-polyacrylamide gel stained with coomassie blue indicating the amounts of recombinant Maf1 and GST used in the *in vitro* transcription experiment described in C. E) Maf1 associates with pol III in the transcription extract. *In vitro* transcription reactions containing either no (lane 2) or 400 ng (lane 3) of recombinant His-tagged Maf1 were incubated with Ni-NTA beads, and the beads were then washed several times with D100 buffer [40]. The affinity-purified complex was analysed by SDS-PAGE followed by immunoblotting with anti-RPC1 or anti-His tag antibodies.

To examine whether human Maf1 is a negative regulator of pol III transcription, we first tested its effects on pol III transcription in a HeLa cell extract. We tested transcription from the 5S type 1 promoter, the Adenovirus 2 (Ad2) VAI type 2 promoter, and the U6 type 3 promoter. We also tested the 7SL promoter, a mixed type 2 promoter with activating elements upstream of the transcription start site [24], as well as the Ad2 major late (ML) promoter, which is recognized by pol II. As shown in Figures 1A and 1C, all templates were efficiently transcribed (compare lanes 1 and 2). However, upon addition of increasing amounts of recombinant Maf1 produced in E. coli, transcription from all types of pol III promoters, but not from the pol II Ad2 ML promoter, was severely depressed (lanes 3–6). In contrast, addition of increasing amounts of similarly prepared Brf2, which is not involved in transcription from type 1 and 2 pol III promoters nor from pol II promoters, did not repress 5S, VAI, 7SL, and ML transcription, and addition of increasing amounts of similarly prepared GST did not decrease U6 transcription (lanes 7–10). Panels B and D show that similar amounts of recombinant Maf1, Brf2, and GST were used in these experiments as determined by coomassie blue-staining of protein gels. Thus, Maf1 efficiently and specifically represses pol III transcription from type 1, 2, and 3 promoters *in vitro*, but not pol II transcription from the Ad2 ML promoter.

In the yeast system, Maf1 can repress pol III transcription *in vitro*, and the *in vitro* effect is attributed mainly to inhibition of TFIIIB-promoter DNA complex assembly, specifically Brf1 recruitment, as well as to inhibition of pol III recruitment [23]. We can monitor assembly of TBP, Brf2, Bdp1, and SNAP_c_ on a U6 promoter by EMSAs [25], and so we checked whether addition of recombinant human Maf1 inhibited formation of either the four-factor complex or various subcomplexes on the U6 promoter. We were unable to detect inhibition of complex formation by Maf1, suggesting that Maf1 inhibition of *in vitro* U6 transcription is not due to effects on transcription factor assembly (data not shown). We tested, therefore, whether recombinant Maf1 could associate with pol III in the HeLa cell transcription extract. We incubated nickel beads in extracts either not supplemented- or supplemented- with His-tagged Maf1, and checked the retained material for the presence of RPC1, the largest subunit of pol III. As shown in Figure 1E, RPC1 was specifically recovered from extracts supplemented with Maf1 (compare lanes 2 and 3). This is consistent with a repression mechanism involving Maf1 association with pol III.

### Endogenous Maf1 associates with pol III and Brf1, and Maf1 can associate with two individual pol III subunits *in vitro*

**Figure 2:**
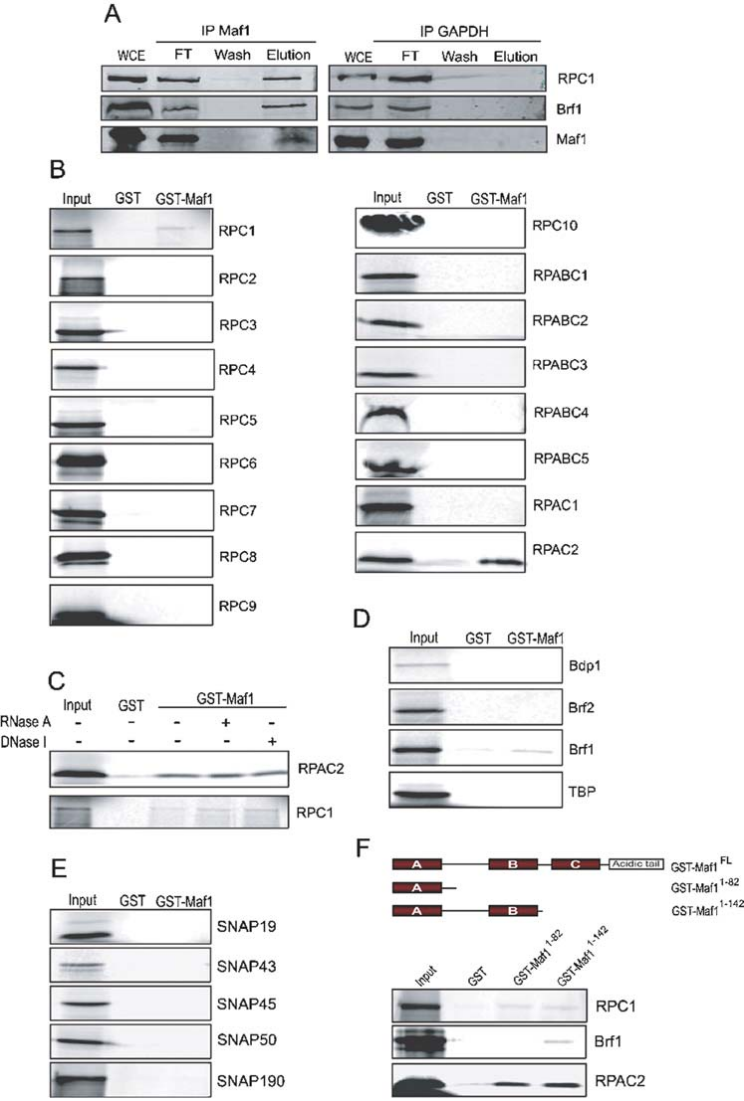
Maf1 associates with pol III, the individual pol III subunits RPC1 and RPAC2, and Brf1. A) Association of endogenous Maf1 with pol III and Brf1. Whole cell extract derived from the HeLa cell line 9–8 [41] was used for immunoprecipitations with anti-Maf1 or anti-GAPDH (Abcam) antibodies. The beads were washed, bound material was then eluted by boiling and used for immunoblotting with an anti-RPC1 antibody (CS377) (upper panel), an anti-Brf1 antibody (CS146) (middle panel) or an anti-Maf1 antibody (SZ2793) (lower panel). Lane 1 shows 1/20 of the input material, lanes 2, 3, and 4 show 1/30 of the flow through, 1/30 of the last wash, and 1/5 of the eluted material, respectively. Quantification of the signal shows that 6% of the RPC1 protein was co-immunoprecipitated with Maf1. B) Association of Maf1 with individual pol III subunits. GST-Maf1 or just GST were expressed in E. coli and immobilized on glutathione sepharose beads. Pol III subunits were synthesized by coupled *in vitro* transcription/translation in the presence of [^35^S] methionine, and incubated with GST-Maf1 or GST beads. The beads were washed extensively and the bound material was analysed by SDS–PAGE and autoradiography. The first lane (input) shows 1/10 of the *in vitro* translated material added to the beads. C) Association of Maf1 with RPC1 and RPAC2 is not mediated by RNA nor DNA. Before the binding reaction, GST, GST-Maf1, and *in vitro* translated RPC1 and RPAC2 were subjected to RNase A or DNase I treatment as indicated above the lanes. D and E) Association of Maf1 with TFIIIB components and SNAP_c_ subunits, respectively. The experiments were performed as in B but with the *in vitro* translated proteins indicated. F) Pol III subunits and Brf1 associate with different Maf1 regions. On top, a schematic representation of full-length and truncated versions of human Maf1 are shown, with the A, B, and C conserved regions indicated. The experiment shown in the three bottom panels was performed as in B but with the GST fusion proteins indicated and *in vitro* translated GST, GST-Maf1^1-82^, or GST-Maf1^1-142^, as indicated.

The observation that Maf1 can associate with pol III when added to a transcription extract prompted us to test whether pol III could be immunoprecipitated with endogenous Maf1. We performed immunoprecipitations from HeLa whole cell extracts with either an anti-Maf1 or, as a control, an anti-GAPDH, antibody. Input material (whole cell extract, WCE), flow-through, wash, and material eluted from the beads were then analyzed by immunoblotting with an antibody directed against RPC1. As shown in Figure 2A, RPC1 was clearly retained on the anti-Maf1, but not the anti-GAPDH, beads, indicating that endogenous Maf1 can associate with pol III. We also checked the precipitated material for the presence of the transcription factor Brf1, because yeast Maf1 has been reported to associate with yeast Brf1 [23]. Indeed, endogenous human Brf1 was specifically present in the anti-Maf1 immunoprecipitate (figure 2A), raising the possibility that it interacts directly with human Maf1.

We then explored the ability of Maf1 to associate with individual pol III subunits and transcription factors. The 17 pol III subunits, as well as the 5 subunits of SNAP_c_, and the Brf1- and Brf2-TFIIIB components Bdp1, Brf2, Brf1, and TBP were all translated *in vitro* in the presence of [^35^S] methionine and tested for association with recombinant GST-Maf1 or GST alone immobilized on beads. As shown in Figure 2, panels B, D, and E, of these 26 proteins, only three were significantly retained on the GST-Maf1 beads, namely the largest pol III subunit RPC1 and the pol I and pol III subunit RPAC2 (AC19) (panel B), as well as Brf1 (panel D). Although the interactions with RPC1 and Brf1 were weak, they were clearly above background. The interactions with the pol III subunits RPC1 and RPAC2 were resistant to treatment with RNase A and DNase I (panel C) and are thus unlikely to be mediated by RNA or DNA. These results are consistent with the associations of yeast Maf1 with the largest subunit of yeast pol III [19] and Brf1 [23] observed previously. They indicate that when components of the human pol III transcription machinery are systematically tested, only one additional polypeptide, RPAC2, associates with Maf1, but this association is by far the strongest one and may thus be responsible for most of the observed association between endogenous Maf1 and pol III (Figure 2A). The lack of association with any of the factors used by type 3 promoters, i.e. all SNAP_c_ subunits, Bdp1, TBP, and Brf2, is consistent with the idea that Maf1 does not affect assembly of these factors on the U6 promoter.

Maf1 contains three region of high phylogenetic sequence conservation designated the A, B, and C boxes, followed by an acidic tail. As shown in Figure 2F, we generated truncated versions of the protein either containing the A box sequence (Maf1^1-81^) or containing both the A and the B box sequences (Maf1^1-142^ ), fused to an N-terminal GST tag, and checked their ability to retain RPC1, Brf1, and RPAC2 in a GST pull-down experiment as above. As before, all three proteins associated with full-length Maf1 (not shown). However, whereas RPC1 and RPAC2 could associate with the Maf1^1–81^ truncated version containing just the A box, Brf1 only associated with the longer Maf1^1–142^ truncated version, containing both the A and the B boxes. Thus, whereas the first 81 amino acids are sufficient for association with pol III subunits, the B box is required for association with Brf1, indicating that Maf1 interacts with pol III and Brf1 via different domains.

### Knock-down of endogenous Maf1 in non-transformed and transformed cells results in higher levels of unstable tRNA precursors

**Figure 3:**
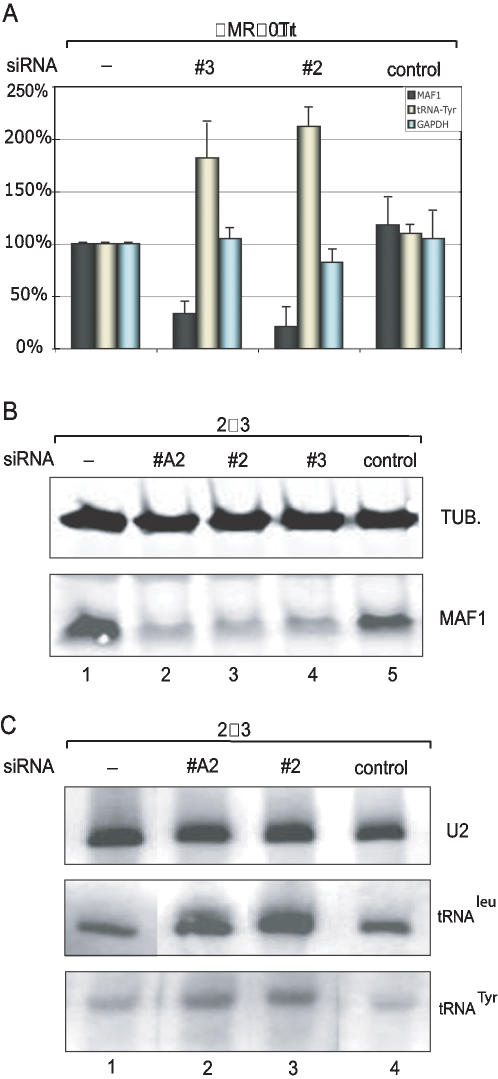
Knock-down of endogenous Maf1 increases the levels of unstable tRNA precursors. A) Knock-down of endogenous Maf1 in untransformed cells. Exponentially growing IMR-90Tert cells were either left untransfected, or transfected with either siRNAs #2 or #3 against MAF1, or with a control siRNA, as indicated on top of the panel. RNA was then isolated from untransfected or transfected cells and 2 µg of total RNA used for random-primed reverse transcription. The resulting cDNA was analyzed by real-time quantitative PCR (qPCR) with primers corresponding to either Maf1 (black bars), tRNA^Tyr^ precursor (yellow bars), or GAPDH as a control (blue bars). B) and C) Knock-down of endogenous Maf1 in transformed cells. In B, the levels of either α-tubulin as a loading control (upper panel) or Maf1 (lower panel) were analyzed by western-blot in extracts from either untransfected 293 cells (lane 1) or 293 cells transfected with the siRNAs indicated above the lanes (lanes 2–5). In C, 293 cells were either left untransfected (lane 1) or transfected with the siRNAs indicated above the lanes. Cellular RNA was then collected and analyzed by northern blot with a probe detecting U2 snRNA precursors as a control (upper panel), or intron-containing tRNA^Leu^ (middle panel) and tRNA^Tyr^ (bottom panel) precursors.

To examine the effects of Maf1 in cultured cells, we used RNA interference (RNAi) to decrease the amounts of endogenous Maf1. We first used a non-transformed human lung fibroblast cell line IMR-90 stably expressing the human Tert protein (IMR-90Tert, a gift from G.J. Hannon, Cold Spring Harbor Laboratory). As shown in Figure 3A, Maf1 mRNA analysis with reverse transcription followed by quantitative real-time polymerase chain reaction (RT-qPCR) showed a severe decrease after transfection with two different silencing RNAs (siRNAs) (# 2 and # 3) directed against Maf1, but not after transfection with a control siRNA (black bars). Strikingly, the levels of the intron-containing unstable tRNA^Tyr^ precursor [26] increased by about two fold (yellow bars), whereas those of the pol II-transcribed Glyceraldehyde-3-phosphate-dehydrogenase (GAPDH) mRNA varied very little (blue bars).

We then examined the effect of reducing levels of Maf1 in the transformed human embryonic kidney (HEK) 293 cells. As shown in Figure 3B, three different siRNAs directed against Maf1 (#A2, #2, and #3), but not a control siRNA, reduced Maf1 protein by more than 80% (compare lanes 2–4 to lanes 1 and 5). A northern blot analysis of total RNA with a probe hybridizing to the intronic regions of the tRNA^Tyr^ and tRNA^Leu^ precursors [26], [27], shown in Figure 3C (middle and bottom panels), revealed that in all cases where Maf1 protein levels were decreased, the levels of tRNA precursors were increased. When normalized for the levels of the pol II U2 snRNA precursor [28](upper panel), which varied little, tRNA precursor levels increased 2 to 2.5 fold. Thus, reducing intracellular levels of Maf1 results in increased levels of precursor tRNAs, consistent with the idea that Maf1 is a repressor of pol III transcription.

### Knock-down of endogenous Maf1 results in higher pol III transcription after MMS treatment

**Figure 4:**
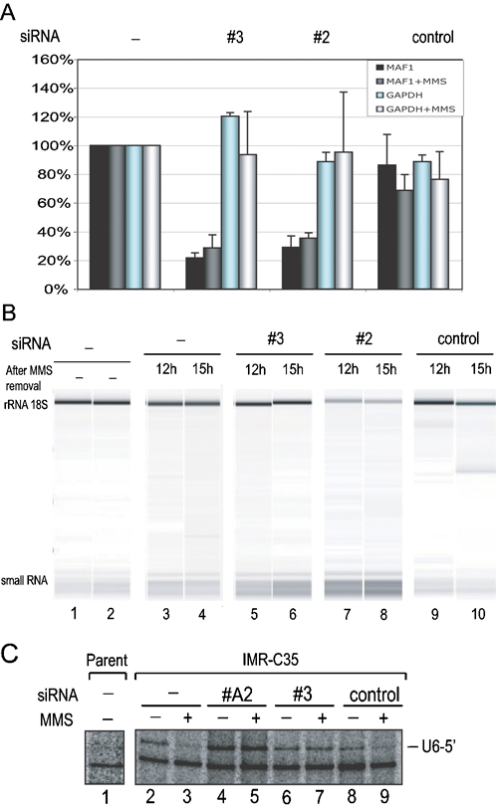
Knock-down of endogenous Maf1 results in higher pol III transcription after stress. A) RT-qPCR analysis of Maf1 and GAPDH RNA before (black and blue bars, respectively), and after (grey and white bars, respectively) MMS treatment (1 mM MMS for 2 hours) in IMR-90Tert cells either left untransfected or transfected with siRNAs against Maf1 (#2 and #3), or control siRNA, as indicated on top of the panel. Total RNA was analyzed as in Figure 3A. B) Total RNA from exponentially growing IMR-90Tert cells either left untransfected or transfected with siRNAs against MAF1 (#2 and #3) or a control siRNA, as indicated on top of the panel, were either not treated (lanes 1, 2) or treated (lanes 3–10) with 1 mM MMS for 2 hours. Samples isolated 12 and 15 h after MMS removal are shown, as indicated (lanes 3–10). Equal amounts of total RNA (∼100 ng/µl) were then resolved on a Bioanalyzer, showing the 18S ribosomal RNA in the top band and short RNAs concentrated in the bottom band. C) Exponentially growing IMR-C35 cells were transfected with siRNA #A2 and #3 against Maf1 or a control siRNA, and then treated or not with 1 mM MMS for 2 hours, as indicated above the lanes. Total RNA was isolated 12–15 hours after MMS removal and 10 µg analyzed by RNase T1 mapping to reveal the transcript derived from the pU6/RA.2+U6end-Dsred integrated construct. Lanes 1 shows a similar RNAse T1 assay performed with RNA from the parental IMR-90Tert cell line. U6-5′: signal corresponding to correctly initiated U6/RA.2+U6end RNA.

In yeast cells, Maf1 is essential for down-regulation of pol III transcription after stress. To examine the role of Maf1 in human cells after stress, we first treated both transformed HEK 293 and non-transformed IMR-90Tert cells with methane methylsulfonate (MMS), an alkylating agent that causes DNA damage, or rapamycin, an antibiotic that inhibits the TOR kinase and mimicks nutrient deprivation (see [29]). However, MMS did not significantly reduce pol III transcription in the transformed HEK 293 cells (data not shown), and we therefore focused on IMR-90Tert cells. Figure 4A shows an RT-qPCR analysis of Maf1 mRNA levels after transfection of these cells with various siRNAs. MMS treatment had little effect on Maf1- (10% decrease) and GAPDH- (20% decrease) mRNA levels, and so these levels are set at 100% in the figure (left: no siRNA) to facilitate visual comparison of the siRNA effects. Transfection of two different siRNAs directed against Maf1 (# 3 and # 2), but not of a control siRNA, decreased Maf1 mRNA levels to less than 20%, both in the absence or presence of MMS (black and grey bars). By comparison, the levels of GAPDH mRNAs varied little, and not in a consistent manner (blue and white bars). We then analyzed a constant amount of total RNA from these cells on a Bioanalyzer (Applied Biosystems), which separates large RNAs from the small RNA population consisting largely of pol III transcripts, in particular mature, stable tRNAs. As shown in Figure 4B, there was little change in the global amounts of small RNAs 12 h and 15 h after MMS treatment in untransfected cells or cells transfected with the control siRNA (lanes 3, 4, 9, and 10). This lack of apparent decrease after MMS treatment probably reflects the great stability of most mature pol III transcripts. In cells transfected with siRNAs directed against Maf1, however, there was an increase in the amounts of small RNAs after MMS treatment (lanes 5–8), as well as after rapamycin treatment (data not shown), consistent with the idea that when Maf1 levels are diminished, pol III transcription continues even after stress.

To confirm the attenuation of pol III repression after MMS treatment, and to determine whether it affected a type 3 promoter, we created an IMR-90Tert cell line containing an integrated construct in which the U6 promoter directs the synthesis of an unstable RNA, whose levels, therefore, better reflect transcriptional activity (see Experimental Procedures). Figure 4C shows an RNAse T1 protection analysis of these U6 promoter-directed transcripts. As expected, they can be detected in the IMR-90Tert U6 reporter cell line, referred to as the IMR-C35 cell line, but not in the parent cell line (compare lane 1 with, for example, lanes 2 and 8). In untransfected cells and cells transfected with the control siRNA, U6 promoter-directed transcription was severely diminished after MMS treatment (compare lane 2 to lane 3, and lane 8 to lane 9), as expected after transcription repression of an unstable RNA. After Maf1 levels were decreased by RNAi, the levels of U6 transcription were either not, or only modestly, increased in the absence of MMS, suggesting that unlike the tRNA promoters analyzed in Figure 3, the much weaker U6 promoter is not repressed by Maf1 in actively dividing cells (compare lanes 2 and 8 to lanes 4 and 6). However, after MMS treatment, the decrease of Maf1 levels prevented repression of U6 transcription (compare lanes 5 and 7 to lanes 3 and 9). Collectively, these results suggest that human Maf1 is required for repression of pol III transcription after DNA damage.

### Maf1 is phosphorylated in mammalian cells

**Figure 5:**
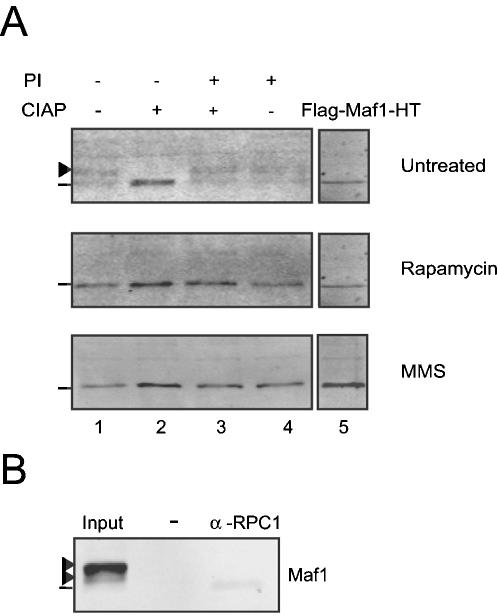
Human Maf1 is phosphorylated and becomes dephosphorylated after rapamycin and MMS treatment. A) Nuclear extract from a HEK 293 cell line expressing HA-tagged Maf1 either untreated (upper panel), or treated with rapamycin (middle panel) or MMS (lower panel) was incubated with nothing, CIAP with or without phosphatase inhibitors, or phosphatase inhibitors alone, as indicated above the lanes. Tagged Maf1 was visualized with an antibody directed against the HA tag. Lane 5 shows bacterially expressed Maf1, which was loaded on a non-adjacent lane of the same gel. B) Nuclear extract from IMR-90Tert cells transiently expressing HA-tagged Maf1 was prepared in the presence of phosphatase inhibitors prior to immunoprecipitation with affinity-purified anti-RPC1 antibody. Tagged Maf1 was visualized with an antibody directed against the tag (α-HA antibody). Only the fastest migrating form of Maf1 bound to pol III. Arrows: phosphorylated Maf1. The negative control was a mock immunoprecipitation performed with protein A sepharose beads without antibody. Lane 1 shows 1/20 of the input material.

Yeast Maf1 is a phosphoprotein that is rapidly dephosphorylated under stress conditions that lead to pol III repression [19], [20]. We therefore tested whether human Maf1 is phosphorylated, and whether its phosphorylation status changes under stress conditions. We subjected a human embryonic kidney 293 stable cell line expressing HA-tagged Maf1 to rapamycin or MMS treatment. Nuclear extract from untreated and treated cells was prepared and incubated with Calf Intestine Alkaline Phosphatase (CIAP), either without or with phosphatase inhibitors. As shown in Figure 5, upper panel, immunoblotting of equal amounts of nuclear extracts with an anti-HA antibody showed several bands, in particular one (indicated by a line) migrating close to recombinant flag-Maf1-HT (lane 5) and a slower migrating tight doublet (indicated by an arrowhead) (lane 1). The tight doublet disappeared while the lower band increased in intensity after treatment with CIAP in the absence, but no the presence, of phosphatase inhibitors (compare lanes 2 and 3). This indicates that the tight doublet is a phosphorylated form of Maf1. Significantly, in extracts from cells treated with rapamycin or MMS, only the fast-migrating form of Maf1 comigrating with recombinant Maf1 was observed (middle and bottom panels). This fast migrating form may be entirely dephosphorylated or may contain phosphate groups that do not affect its migration. Similar results were obtained with whole cell rather than nuclear extracts (data not shown).

We then proceeded to determine whether Maf1 is phosphorylated in the non-transformed IMR-90Tert cells, and which form of Maf1 binds to pol III. We prepared nuclear extracts from IMR-90Tert cells expressing HA-tagged Maf1. To avoid partial dephosphorylation of Maf1, we prepared the extract in the presence of phosphatase inhibitors. The extract was then used as the starting material in an immunoprecipitation with an anti-RPC1 antibody, and the immmunoprecipitate was probed for the presence of Maf1. As shown in Figure 5B, several forms of Maf1 were present in the starting material, and only the minor fastest migrating form was found in the anti-RPC1 immunoprecipitate. Together, the data indicate that tagged Maf1 is phosphorylated in human cells, that it is at least partially dephosphorylated under stress conditions, and that it is the dephosphorylated form that associates with pol III. Thus, pol III repression and association with pol III are linked to Maf1 dephosphorylation in human cells.

## Discussion

Human Maf1 is a repressor of pol III transcription, as suggested by the observations that it represses pol III transcription *in vitro* when added to transcription reactions (Figures 1A and 1C) and that its knock-down in cells results in higher levels of short-lived tRNA precursors (Figure 3). This last result, observed in actively dividing cells, suggests that in such cells, there is some Maf1 activity that keeps pol III transcription, in particular from tRNA promoters, in check, consistent with the observation in yeast that some Maf1 is nuclear and active even when the cells are in exponential phase [22]. On the other hand, weaker promoters such as the U6 promoter may not be partially repressed in actively dividing cells (Figure 4C).

In yeast, Maf1 represses transcription by at least two mechanisms: by acting on pol III itself, probably already bound to promoters [19], [20], and by preventing assembly of Brf1 into initiation complexes [23]. We could not see any effects of Maf1 in assembly of SNAP_c_, TBP, Brf2 and Bdp1 on the type 3 human U6 promoter. Moreover, Maf1 did not associate with any of these proteins in a GST pull-down assay (Figure 2). We could, however, detect association of exogenously added Maf1 with pol III under *in vitro* transcription conditions (Figure 1E), association of endogenous Maf1 and pol III (Figure 2A), and association with two individual pol III subunits in the GST pull-down assay (Figure 2B). This suggests that at least for type 3 promoters, where we tested assembly of all the factors required to recruit a pol III complex, Maf1 does not act on formation of the promoter-bound pol III-recruiting complex but rather by binding to pol III. On the other hand, we detected association of Maf1 with endogenous Brf1, as well as a weak association in the GST pull-down assay. This association parallels that observed with the yeast protein [23] and suggests that Maf1 may have an effect on formation of the pol III-recruiting complex on human type 1 and 2 promoters, which use Brf1- rather than Brf2- TFIIIB. Interestingly, the Maf1 region required for association with Brf1 is different from that required for association with pol III subunits.

Yeast Maf1 interacts genetically with the E. coli β′-like largest subunit of pol III [17], and this has prompted the testing of whether the two proteins associate with each other. Indeed, Maf1 associates with the first 235 amino acids of C160/RPC1 [19]. However, at least for human Maf1, a systematic testing of all the human pol III subunits indicates a much stronger interaction with the E. coli α-like subunit AC19/RPAC2 as compared to RPC1 (Figure 2B). A superposition of the human RPC1 and RPAC2 sequences on those of the paralogue pol II subunits RPB1 and RPB11 in the pol II crystal structure [30] indicates that, as expected for subunits corresponding to the E. coli RNA polymerase β′- and α-like subunits, the two polypeptides contact each other. Nevertheless, the first 235 amino acids of RPC1 are not close to RPAC2. Thus, if as in yeast, the RPC1 association with Maf1 is through the first 235 RPC1 amino acids, it is not clear that RPC1 and RPAC2 are contacted at the same time.

Maf1 is involved in at least two repression pathways in human cells, the MMS and rapamycin pathways, because knock-down of Maf1 diminishes pol III repression after these treatments (Figure 4 and data not shown). Interestingly, Maf1 is active in transformed 293 cells, since knock-down of Maf1 in these cells increases pol III transcription (Figure 3), even though we find that MMS treatment does not lead to the rapid pol III repression seen in IMR-90Tert cells (data not shown). This indicates that even though some signaling pathways to Maf1 are lost in these cells, the Maf1 protein itself is still active. Intriguingly, human Maf1 is phosphorylated in 293 cells and phosphorylation is lost after both MMS and rapamycin treatments (Figure 5A). In yeast, Maf1 dephosphorylation is linked to nuclear localization, but nuclear localization is not sufficient for transcription repression, indicating that other signals are necessary to activate the protein [22]. This suggests that in 293 cells, Maf1 may localize correctly to the nucleus after MMS treatment but that another signal, lost in 293 cells, is required for Maf1 activation by DNA damage. Maf1 could potentially be a tumor suppressor. It will be interesting to determine whether in some other tumor cells, the Maf1 protein itself, rather than signaling to Maf1, is debilitated.

## Materials and Methods

### Plasmids and constructs

The Maf1 coding region from a human cDNA encoding full-length human Maf1 (GenBank accession number NM_032272) or Maf1^1-81^ and Maf1^1-142^ fragments were amplified by PCR using specific primers and inserted into various vectors. For expression in mammalian cells as N-terminal HA tag fusions, it was inserted into the pCGN vector [31]. For expression in E. coli as an N-terminal Flag and C-terminal His tag fusion, or as an N-terminal GST fusion, it was inserted into pSBet [32] derivatives. All clones were verified by DNA sequencing. pU6/Hae/RA.2 [33], T3/T7 H7L30.1 (carrying the 7SL gene) [24], pH5SST [34], pBSM13+VA1 [35], and p119MLP(C2A) carrying the Ad2 major late promoter [35] were described previously. pU6/RA.2+U6end-Dsred is a derivative of the pDsRed-Express-DR vector (Clontech) with an insert consisting of the human U6 promoter followed by a cassette corresponding to a piece of β-globin mRNA cloned in the reverse orientation as in pU6/Hae/RA.2 [33], itself followed by the natural U6 3′ end and 3′ flanking sequences.

For *in vitro* transcription/translation, we used the pCite-2a(+) (Novagen) derived plasmids pNCite/RPC1, pNCite/HsRPC2, pCite/HsRPC62/HA (expressing RPC3), pNCiteBN51 (expressing RPC4), pCite/hu75k3 (expressing RPC5), pCite/RPC39/Flag (expressing RPC6), pCite/HsRPC32/HA (expressing RPC7), pNCite/HsRPC25 (expressing RPC8), pCite/HsCGRP (expressing RPC9), pNCite/HsRPC10stop, pNCite/RPAC1/stop, pNCite/RPAC2, pNCite/HsRPABC2, pNCite/HsRPABC3, pNCite/HsRPABC5, pM3/190III Stop/Bam (expressing SNAP190), pNCite/SNAP50, pNCite/SNAP45, pCite 43-1 (expressing SNAP43), pCite/SNAP19, pCite/B″short#11 (expressing Bdp1), pCite/Brf2, pNCite/Brf1, pCite/hTBP, and pSBet-derived plasmids pSB/Flag/HsRPABC1stop and pSB/flag/RPB12 (expressing RPABC4).

### Glutathione S-transferase (GST) pull-down assays

Recombinant GST, GST-Maf1, GST-Maf1^1-81^ or GST-Maf1^1-142^ were expressed in E. coli BL21 DE3 cells with the T7 system from Studier et al. [36]. The proteins were purified on glutathione Sepharose beads in the presence of 200 µg of RNase A (Sigma) or 5 U/ml of DNase I (Ambion) and the beads were then incubated with [^35^S] methionine-labeled proteins obtained by coupled *in vitro* transcription/translation in rabbit reticulocyte lysate (TNT® T7 Quick Coupled Transcription/Translation System from Promega). Each binding reaction contained ∼36 µg of GST or ∼70 µg of GST fusion protein immobilized on beads and (∼3.5–4.1 ng) of radiolabeled *in vitro* translated protein. The reactions were incubated in phosphate-buffered saline (PBS) supplemented with 0.05% NP40 at 4°C for 2 h with constant mixing on a wheel. The beads were then washed with the same buffer and the bound (and input) material fractionated on an SDS–polyacrylamide gel and detected with a Typhoon PhosphorImager (Amersham). In Figure 2B, GST, GST-Maf1, and the [^35^S] methionine-radiolabeled *in vitro* translated protein were treated with 100 µg of RNase A (Sigma) or 4 U of DNase I (Ambion) for 20 min at 30°C prior to the binding reaction. Proteins were resolved on 12% high-TEMED SDS–polyacrylamide gels.

### Cell culture and transfection

Human HEK 293 and IMR-90Tert cells were maintained in Dulbecco's modified Eagle medium (DMEM; GIBCO) supplemented with 10% fetal bovine serum, 100 U/ml penicillin, 100 µg/ml streptomycin, and, for the IMR-90Tert cells, 0.1 mM of non-essential amino acids.

To establish a stable human embryonic kidney 293 cell line expressing HA-tagged Maf1, 293 cells were grown to 50–60% confluency in 10 cm dishes and transfected with 1 µg of pCGN-Maf1 and 100 ng of pY3 (a plasmid conferring hygromycin resistance) complexed with Lipofectamine 2000 (LifeTechnologies). The cells were split 24 h later and kept under hygromycin selection (600 µg/ml) for 21 days. Individual clones were then expanded and tested for HA-Maf1 expression.

To establish the IMR90-C35 cell line expressing a U6 promoter-directed unstable RNA, IMR-90Tert cells were transfected by the calcium phosphate method with 5 µg of pU6/RA.2+U6end-Dsred. The cells were split 48h later and kept under G418 selection (500 µg/ml) for 21 days. Individual clones were then expanded and tested for expression of the U6 construct. The resulting clonal cell line used here is called the IMR-C35 cell line.

### Antibodies and immunoprecipitations

For immunoprecipitations, we used polyclonal antibodies against RPC1 (CS377), RPC4 (CS682), Maf1 (Ab SZ2793p), GAPDH (Abcam, ab9482) or against the HA tag (clone 12CA5, Roche). RPC1 or RPC4 proteins were detected in western blots by indirect immunostaining with species-specific antibodies (anti-mouse or anti-rabbit, respectively) coupled to Alexa fluorophore (Molecular Probe). HA-tagged proteins were detected directly with anti-HA antibody (clone 3F10, Roche) coupled to HRP.

### *In vitro* transcription assay

VAI, 7SL and S5 transcription reactions were performed as described previously [35] in 10 mM HEPES pH 7.9, 5% glycerol, 50 mM KCl, 0.1 mM EDTA, 1 mM spermidine (Sigma), 1 mM DTT, 5 mM MgCl_2_, 1 mM each ATP, UTP, GTP, and 10 µC of [α-^32^P] CTP (800 Ci/mmol), in a total reaction volume of 20 µl containing 250 ng of pBSM13+VA1, T3/T7 H7L30.1, or pH5SST supercoiled template and 20 to 30 µg of whole-cell extract. Where indicated, increasing amounts of GST, Maf1, or Brf2 were added to the reaction together with the HeLa extract.

U6 *In vitro* transcription reactions were performed as described [37] in a total volume of 40 µl containing 100 ng of pU6/Hae/RA.2, 250 ng of poly (dG-dC)•(dG-dC), 2 µl of ATP mix (0.3 M ATP, 10 µg of phosphocreatine kinase per ml and 10 mM creatine kinase) and 4 µl of HeLa whole-cell extract. The resulting RNAs were analyzed by RNase T1 protection and fractionation on 6% polyacrylamide-urea gel. Ad2 ML transcription reactions were performed as described [35]. 400 ng of supercoiled DNA template was transcribed in a total volume of 30 µl containing 1% PEG_8000_, 1.2 mM O-methyl GTP, 10 mM MgCl_2_, 1U of RNase T1, 1 mM DTT, 1 mM spermidine, 240 µM ATP and UTP, 0.5 µl of α-^32^P CTP, and 30 µg of whole cell extract.

### siRNA transfection and MMS treatment

siRNA oligos were designed and synthesized by Qiagen. IMR-90Tert and HEK 293 cells were seeded at 5–8×10^5^ cells per 10 cm plates the day before transfection. 15 µl of INTERFERin transfection reagent (Polyplus) was added to 400 µl of DMEM serum-free medium containing 20 nM of each siRNA oligo, incubated for 10 minutes, and added to the 10 cm plate containing 4 ml of medium. The Negative Control siRNA Alexa fluor-488 (Qiagen) was used as a control and as a marker for transfection efficiency. 36 to 48 hours after transfection, cells were treated with 1 mM MMS for 2 hours followed by three washes, and resuspended in complete DMEM medium. RNA was isolated at different times after MMS treatment as indicated in the figure legends with the TRIzol reagent (Invitrogen) according to the manufacturer's protocol, and used for Northern blot or RNase T1 analysis. Radioactivity was measured on a Typhoon Trio+ imaging system and quantified with the ImageQuant software (Amersham Biosciences).

### Quantitative Polymerase Chain Reaction (qPCR)

RNA was quantified with a NanaDrop instrument (Nanodrop technologies) and 2 µg were used for reverse transcription with random hexamer primers in the Improm-II reverse transcription system (Promega). 2 µl of the resulting cDNA was amplified with 0.4 µM of 1) the forward primer TGCCCACATCATTGGCAGGATTG and the reverse primer TGAGCGTGGCAATCAGGTAGAAGA to produce a 237 bp long Maf1 fragment; 2) the forward primer CCTTCGATAGCTCAGCTGGT and reverse primer GTCCACAAATGTTTCTACAGG to produce a 58 bp long tRNA^Tyr^ precursor fragment; 3) the forward primer CATGTTCGTCATGGGTGTGAACCA and the reverse primer AGTGATGGCATGGACTGTGGTCAT to produce a 160 bp long GAPDH fragment. The reactions containing the SensiMix SybrGreen amplification system (Quantace) were set up with a Cas-1200 pipetting robot and analyzed by quantitative PCR on a Rotor-Gene-3000 (Corbett, life science). The thermal cycling conditions were optimized according to the manufacturer's protocol. The results were analyzed with the software provided with the instrument, using the comparative quantification function. The quantification was normalized relative to PCRs performed with cDNA from untreated cells.

### Northern-Blot

10–20 µg of RNA was fractionated on a 7% denaturing polyacrylamide gel and transferred onto a Hybond-N+ nylon membrane (Amersham) with the BioRad PAGE and transfer systems. The membrane was incubated in pre-hybridization buffer containing 500 mM NaHPO_4_ (pH 7.2), 7% SDS, and 5 mM EDTA at 80°C during 2–4 hours. About 2×10^8^ cpm of radioactively labeled probe were then added to the prehybridization buffer and the incubation was continued overnight at 50 and 60°C for the tRNA and U2 probes, respectively. The resulting radioactive signals were visualized with a Typhoon Trio+ imaging system and quantified with ImageQuant (Amersham Biosciences).

### Dephosphorylation assay

Nuclear extract was prepared from the 293 cell line expressing the HA-Maf1 fusion protein either untreated or treated with 0.2 µg/ml of Rapamycin for 16 h or 0.5 mM MMS for 2 h. The cells were incubated in lysis buffer (10 mM HEPES [pH 7.9], 100 mM KCl, 1 mM EDTA, 1 mM DTT, and 0.5% NP-40) for 4 min at 4°C, mixed vigorously, and centrifuged at 5000 rpm for 1 min at 4°C. The nuclear pellets were washed with lysis buffer lacking NP-40, resuspended in 250 mM Tris–HCl [pH 7.9], 100 mM KCl, 1 mM EDTA, 1 mM DTT, 0.5% NP-40, 20% glycerol and mixed on a wheel at 4°C for 30 min. The extract was then centrifuged at 10 000 rpm for 30 min at 4°C, the supernatant collected and used directly [38]. Alternatively, whole cell extract was prepared as described before [39]. 20 µl of nuclear or whole cell extract was treated with 10 U of Calf Intestine Alkaline Phosphatase (CIAP) (Roche) for 30 min at 37°C in 50 mM Tris-HCl (pH 8.0) and 0.1 mM ZnCl_2_. Phosphatase inhibitors (1.5 mM p-NO_2_-phenyl-phosphate, 1.5 mM sodium fluoride and 1 mM sodium orthovanadate) were added as indicated in Figure 5. The proteins were then fractionated by 12% SDS-PAGE and tagged Maf1 detected by immunoblotting with an anti-HA antibody.

Human IMR-90Tert were grown to 50–60% confluency in 10 cm dishes and transfected with 10 µg of the pCGN-derived plasmids complexed with Lipofectamine 2000 (Life Technologies). After 48 h, the cells were treated with 0.2 µg/ml of rapamycin and harvested 72 h post transfection. Small-scale nuclear extract preparation and immunoprecipitations were performed as above except that the IMR-90Tert nuclear extract was treated with 10 U of DNase and phosphatase inhibitors (10 mM Na pyrophosphate, 1 mM Na vanadate and 10 mM Na fluoride). Proteins were resolved on a 12% high-TEMED SDS–polyacrylamide gel, transferred to a membrane, and analysed by western blots with anti-HA conjugated to Alexa800 fluorophore (Rockland).
